# Medium Chain Triglyceride (MCT) Oil Affects the Immunophenotype via Reprogramming of Mitochondrial Respiration in Murine Macrophages

**DOI:** 10.3390/foods8110553

**Published:** 2019-11-05

**Authors:** Seungmin Yu, Gwang-woong Go, Wooki Kim

**Affiliations:** 1 Department of Food Science and Biotechnology, Graduate School of Biotechnology, Kyung Hee University, Yongin 17104, Korea; dbtmdals1004@khu.ac.kr; 2 Department of Food and Nutrition, Hanyang University, Seoul 04763, Korea

**Keywords:** medium chain triglyceride (MCT), mitochondrial metabolic reprogramming, macrophage polarization

## Abstract

Medium chain triglyceride (MCT) oil has been postulated to modulate inflammatory responses, but the detailed mechanisms have not been fully elucidated. Based on recent studies demonstrating that mitochondrial metabolic reprogramming and immune responses are correlated, the current study sought to determine whether MCT oil controls inflammatory responses through modulation of mitochondria using both in vitro and in vivo models. The mitochondrial metabolic phenotypes of macrophages were assessed according to oxygen consumption rate (OCR). Inflammatory responses were assessed for production of cytokines and expression of activation markers. MCT oil was more rapidly oxidized as observed by increased OCR in macrophages. The production of pro-inflammatory cytokines was down-regulated and anti-inflammatory cytokine was elevated by MCT oil. In addition, classically activated M1 and alternatively activated M2 markers were reciprocally regulated by MCT intervention. Overall, up-regulated β-oxidation by MCT contributes to the anti-inflammatory M2-like status of macrophages, which may aid in the dietary prevention and/or amelioration of inflammation.

## 1. Introduction

A number of recent studies have demonstrated that mitochondrial energy metabolism has a strong correlation with inflammatory responses, including macrophage polarization [1,2]. Specifically, classically activated M1 macrophages depend on glycolysis and further lactate fermentation, even in the presence of excess oxygen in cytoplasm. In contrast, alternatively activated M2 macrophages seem to utilize oxidative phosphorylation (OXPHOS), for which fatty acids from dietary lipids might aid in the supply of substrate acetyl-CoA through β-oxidation [3].

An extracellular flux analyzer was recently developed to quantify glycolysis and OXPHOS by real-time assessments of proton efflux and oxygen consumption, respectively, in the micromilieu of the cellular environment [4]. In accordance with the above-mentioned cellular energy metabolism, murine bone-marrow derived macrophages (non-polarized, M0) were successfully demonstrated to reciprocally regulate oxygen consumption in lipopolysaccharide (LPS) stimulated M1 vs. interleukin (IL)-4/IL-13 induced M2 subsets [5].

Dietary lipids are one of the major energy suppliers for cellular survival, and many studies have shown that the fatty acid compositions of lipids dictate their physicochemical and/or physiological functions. In this regard, long-chain fatty acids abundant in conventional dietary lipids require a carrier enzyme, carnitine acyltransferase (CAT), to migrate into the mitochondria for β-oxidation [6]. In contrast, medium-chain fatty acids directly penetrate the mitochondrial inner membrane in a CAT-independent manner partially due to lower hydrophobicity. Subsequently, previous studies have shown that medium-chain fatty acids are rapidly metabolized to acetyl-CoA, resulting in increased cellular energy expenditure and reduced body fat accumulation compared to long-chain fatty acids [7,8]. In addition, the substitution of dietary long-chain fatty acids with medium-chains exhibited anti-inflammatory effects by down-regulating pro-inflammatory genes and cytokines in LPS-induced M1 macrophages, yet the detailed mechanisms underlying these phenomena are not fully understood [9].

As an extension to previous observations, in the current study it was hypothesized that dietary supplementation with medium-chain fatty acids in the form of TG up-regulates OXPHOS in macrophages. For this purpose, RAW 264.7 murine macrophages or C57BL/6 male mice were given MCT oil in culture medium or feed pellets, respectively. Following the MCT oil intervention, M1/M2 macrophages were assessed for polarization, cellular energy metabolism using either glycolysis or OXPHOS, and pro-/anti-inflammatory cytokine production.

## 2. Materials and Methods

### 2.1. Cell Culture and Oil Treatments

The murine macrophage RAW 264.7 cell line was purchased from the Korea Cell Line Bank (KCLB, Seoul, Korea). The cells were seeded on 24-well plates at a concentration of 1 × 10^5^ cells/mL in DMEM supplemented with 10% FBS and 1% antibiotic/antimycotic solution (10,000 U/mL penicillin G, 10,000 μg/mL streptomycin, 25 μg/mL amphotericin B). Medium chain fatty acid-triglyceride (MCT) enriched oil was purchased from Nisshin Oillio (Healthy Resetta™, Tokyo, Japan), and canola oil purchased from CJ CheilJedang (Seoul, Korea) served as a control. MCT and control oils were dissolved in DMSO (Daejung Chemicals, Siheung, Korea) and the RAW 264.7 cells were treated for 24 h at 37 °C in a 5% CO_2_ incubator. The final concentration of DMSO in the cell culture medium was maintained under 0.01% (*v*/*v*). Following oil intervention in medium, macrophages were stimulated by incubating them with 500 ng/mL of TLR ligand approved LPS from *Escherichia coli* O111:B3 (Sigma-Aldrich, St. Louis, MO, USA) for 12 h [10].

### 2.2. Animals and Dietary Interventions

C57BL/6 male mice at the age of 4 weeks were purchased from Raon Bio (Yongin, Korea). The care, use, and treatment of all mice in this study were conducted in strict accordance with guidelines from the Institutional Animal Care and Use Committees of Kyung Hee University. Custom-made mouse diets were purchased from Raon Bio (Yongin, Korea), with the control AIN-76A diet modified by replacement of corn oil with 80% (MCT high diet) or 20% MCT oil (MCT low diet) (Table 1). Following 1 week of mouse acclimation by feeding the AIN-76A diet, mice were randomly separated into three groups (*n* = 7) and fed either MCT high, MCT low, or control AIN-76A diets for 4 weeks ad libitum. After dietary interventions, mice were sacrificed in a CO_2_ chamber.

### 2.3. Bone Marrow-Derived Macrophage (BMDM) Culture and Macrophage Polarization

Mouse mononuclear cells were collected from the femurs and tibias of C57BL/6 mice and treated with NH_4_Cl solutions (Stemcell Technology, Canada) on ice for 10 min to lyse the red blood cells. Mature bone marrow-derived macrophages (BMDMs, M0) were obtained from differentiated mononuclear cells stimulated with 10 ng/mL macrophage colony-stimulating factor (M-CSF, Sigma-Aldrich, St. Louis, MO, USA) for 7 days. BMDMs were further cultured in Iscove’s modified Dulbecco’s medium (IMDM) supplemented with 10% FBS at 37 °C in a 5% CO_2_ incubator for macrophage polarization as follows: 100 ng/mL LPS with 50 ng/mL interferon (INF)-γ for M1 polarization or 10 ng/mL interleukin (IL)-4 with 10 ng/mL IL-13 for M2 activation were used to treat mature BMDMs for 24 h [11].

### 2.4. Measurement of Oxygen Consumption Rate

The oxygen consumption rate (OCR) was measured using a Seahorse Bioscience XF extracellular flux analyzer (Agilent Technologies, Palo Alto, CA, USA) according to the manufacturer’s instructions. Oil treated RAW 264.7 cells and polarized BMDMs were seeded in XF cell culture plates with non-buffered DMEM supplemented with 4500 mg/L glucose, 4 mM glutamine, and 1 mM sodium pyruvate. Then, the plate was incubated for 1 h at 37 °C in a non-CO_2_ incubator and sequentially treated with 1 μM oligomycin (ATP synthase inhibitor), 0.5 μM carbonyl cyanide-4(trifluoromethoxy)phenylhydrazone (FCCP, uncoupling agent of mitochondrial respiration), and 0.5 μM rotenone/antimycin A mix (inhibitor of complex I and III of electron transport chain, respectively) for measurements of basal and maximal respiration and ATP production through assessing changes in oxygen consumption rate in real time. Basal and maximal respiration and ATP production were calculated by the following equations:

Basal respiration: Last oxygen consumption rates before oligomycin injection—non-mitochondrial oxygen consumption rates

Maximal respiration: Maximum oxygen consumption rates after FCCP injection—non-mitochondrial oxygen consumption rates

ATP production: basal oxygen consumption rates—minimum oxygen consumption rates after oligomycin injection

### 2.5. Assessments of Macrophage Activation Markers

The surface marker expressions of RAW 264.7 cells and BMDMs were determined by flow cytometric analysis following staining with specific antibodies conjugated with fluorescences. Briefly, LPS treated RAW 264.7 cells and differentiated BMDMs were washed with cold PBS with 2% FBS followed by incubation with anti-mouse CD16/CD32 mAbs (Fc block, eBioscience, San Diego, CA, USA) for 15 min at 4 °C to prohibit non-specific antibody binding. Without removing Fc block, fluorescence-conjugated mAbs, i.e., fluorescein isothiocyanate-conjugated anti-mouse CD80 (aCD80-FITC, eBioscience, San Diego, CA, USA), phycoerythrin-conjugated anti-mouse CD86 (aCD86-PE), or allophycocyanin-conjugated anti-mouse MHC-II (aMHC-II-APC, eBioscience, San Diego, CA, USA) were used to treat RAW 264.7 cells 10 min at 4 °C. Similarly, BMDMs were stained by fluorescein isothiocyanate-conjugated anti-mouse F4/80 (aF4/80-FITC), phycoerythrin-conjugated anti-mouse CD206 (aCD206-PE, eBioscience, San Diego, CA, USA), peridinin-chlorophyll proteins-cyanin5.5-conjugated anti-mouse CD11b (aCD11b-PerCP-Cy5.5, eBioscience, San Diego, CA, USA), or allophycocyanin-conjugated anti-mouse CD11c (aCD11c-APC, eBioscience, San Diego, CA, USA). Following surface staining with specific antibodies, cells were washed twice with ice-cold PBS and resuspended in 100 μL PBS, then analyzed by a BD Accuri C6 flow cytometer (BD Biosciences, San Jose, CA, USA). F4/80^+^CD11b^+^ population BMDMs were gated and designated as macrophages. The relative expressions of activation markers, i.e., CD80, CD86, and MHC-II for RAW 264.7 cells and F4/80, CD206, CD11b, and CD11c for BMDMs were determined by mean fluorescence intensity (MFI) as quantified by FlowJo^®^ software (BD Biosciences, San Jose, CA, USA).

### 2.6. Cytokine Quantification

Following stimulation of RAW 264.7 cells with LPS or polarization of BMDMs, pro-inflammatory (interleukin (IL)-6 and tumor necrosis factor (TNF)-α) or anti-inflammatory cytokines (IL-10) in the culture medium were quantified by enzyme-linked immunosorbent assay (ELISA) according to the manufacturer’s instructions (BD Biosciences, San Jose, CA, USA). In brief, 96-well plates were coated with capture antibodies overnight at 4 °C. Then, excess antibodies were washed with assay diluent. Cell culture supernatants and each cytokine standard were aliquoted into each well at designated concentrations. After incubating at room temperature and washing with the provided buffer, the captured cytokines were incubated with biotinylated detection antibodies and streptavidin-horse radish peroxidase conjugates (sAv-HRP) at room temperature. Following a series of washing steps, substrate solution was added for enzymatic reactions, and a stop solution (1M H_3_PO_4_) was applied. After the final reaction steps, the absorbance was measured with a microplate reader (Bio-Rad, Hercules, CA, USA) at a 450-nm wavelength.

### 2.7. Statistical Analysis

Data are expressed as mean ± standard error of the mean (SEM). Statistical significance was analyzed by one-way analysis of variance (ANOVA), followed by a post-hoc Tukey’s multiple comparison test using Prism software (GraphPad Software, La Jolla, CA, USA). Significant differences were indicated with different letters in the set of data (*n* = 3–7), with significance identified at *p* < 0.05. Non significance was denoted as N.S.

### 2.8. Ethics Statement

The animal experiments were conducted with the approval of the Institutional Animal Care and Use Committee of Kyung Hee University (approval ID: KHUASP(GC)-17-030).

## 3. Results

### 3.1. MCT Oil Up-Regulates Mitochondrial Respiration in Macrophages

**Figure 1:**
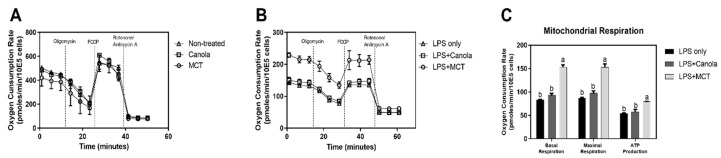
Medium chain triglyceride (MCT) exacerbates mitochondrial oxygen consumption in RAW 264.7 cells. The oxygen consumption rate (OCR) was measured as a marker of oxidative phosphorylation (OXPHOS) using a Seahorse Extracellular Flux analyzer. Samples were initially treated with oils under non-stimulated (**A**) and lipopolysaccharide (LPS)-stimulated conditions (**B**). During extracellular flux analysis, cells were sequentially treated with oligomycin, carbonyl cyanide-4-(trifluoromethoxy)phenylhydrazone (FCCP), and rotenone with antimycin A to assess OXPHOS phenotype based on OCR levels. Based on OCR data (B), the basal respiration, maximal respiration, and ATP production were calculated and are indicated as OCR in pmoles/min (**C**). Data are presented as mean ± SEM (*n* = 4), and significant differences are indicated with different letters (*p* < 0.05) within each basal respiration, maximal respiration, and ATP production column.

In an in vitro model using a RAW 264.7 murine macrophage cell-line, canola oil, known for its anti-oxidant and anti-inflammatory effects which inhibit the production of nitric oxide and prostaglandin E_2_ [12], served as a control. Cytotoxicity of canola or MCT oil emulsified in DMSO, as well as the vehicle itself, was determined by MTT assay. Neither oil affected cell viability up to 100 μg/mL (data not shown), and the oil concentration was set to 10 μg/mL thereafter through the study. In non-stimulated conditions, OCR of mitochondria were not more strongly affected by MCT oil compared to canola oil and non-oil-treated control (Figure 1A). However, following an onset of inflammatory cues by LPS treatment, there was a significant increase of mitochondrial respiration in MCT treated cells (Figure 1B, circle), compared to canola (square) or non-oil-treated (triangle) controls. Throughout the OCR measurements, oligomycin, FCCP, and rotenone/antimycin A were sequentially injected to specifically calculate the cellular oxygen needed for basal respiration, maximal respiration, and ATP production [13]. As quantitatively assessed, MCT oil significantly up-regulated OCR for basal respiration (153.58 ± 4.57 pmoles/min/10E5 cells), maximal respiration (153 ± 7.15 pmoles/min/10E5 cells), and ATP production (79.62 ± 0.26 pmoles/min/10E5 cells), while canola (93.03 ± 3.88 pmoles/min/10E5 cells for basal respiration, 97.91 ± 4.28 pmoles/min/10E5 cells for maximal respiration, and 57.97 ± 4.75 pmoles/min/10E5 cells for ATP production) and non-oil-treated (83.29 ± 0.63 pmoles/min/10E5 cells for basal respiration, 86.78 ± 1.65 pmoles/min/10E5 cells for maximal respiration and 54.48 ± 0.79 pmoles/min/10E5 cells for ATP production) controls exhibited no differences (Figure 1C).

**Figure 2:**
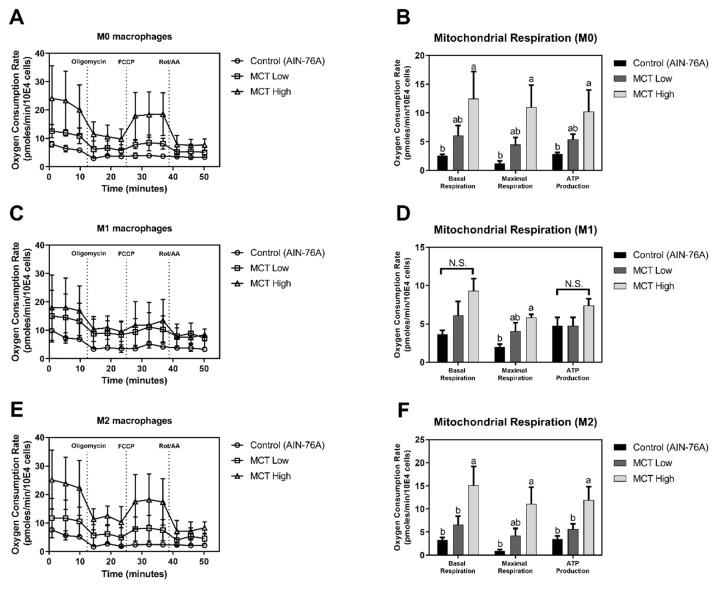
MCT upregulates OXPHOS in non-polarized and M2 polarized BMDMs. To assess OCR in non-polarized (M0) and polarized (M1 and M2) conditions, bone marrow-derived macrophages were collected from C57BL/6 mice fed with custom diets (AIN-76A, MCT Low, MCT High) and treated with macrophage colony-stimulating factor (M-CSF). Following stimulation with LPS/IFN-γ for M1 polarization or interleukin (IL)-4/IL-13 for M2 polarization, cells were transferred to Seahorse microplates for assessing OCR. OCR was assessed using a Seahorse Extracellular Flux analyzer in non-polarized (**A**), M1 (**C**), and M2 (**E**) polarized conditions. The mitochondrial respiration parameters (basal respiration, maximal respiration, and ATP production) were calculated and are presented as pmoles/min in each non-polarized (**B**), M1 (**D**), and M2 (**F**) polarized BMDMs. Data are indicated as mean ± SEM (*n* = 4), and significant differences are indicated with different letters (*p* < 0.05, N.S. means no significant difference) within each basal respiration, maximal respiration, and ATP production column (B, D, and F).

As an in vivo model of MCT-induced regulation of energy metabolism in macrophages, M1/M2 polarized BMDMs isolated from C57BL/6 mice following dietary interventions were examined to assess the mitochondrial consumption of oxygen. The defined AIN-76A diet was used as a control normal diet, while either 20% (MCT low) or 80% (MCT high) corn oil was replaced with MCT oil. The body weight gain exhibited no difference among the diet groups indicating the isocaloric diet uptake in mice (Supplementary Figure S1). In non-polarized (M0) BMDMs, MCT oil-fed groups exhibited significantly up-regulated mitochondrial oxygen consumption compared to the normal AIN-76A diet-fed group (Figure 2A). In accordance to the RAW 264.7 cellular model, dietary provision of MCT resulted in dose-dependent increase of OCR. In detail, MCT high-fed mice exhibited significantly increased OCR for basal respiration (12.47 ± 4.7 pmoles/min/10E4 cells), maximal respiration (11 ± 3.87 pmoles/min/10E4 cells), and ATP production (10.26 ± 3.72 pmoles/min/10E4 cells) in M0 BMDMs compared to normal diet-fed mice (2.54 ± 0.28 pmoles/min/10E4 cells for basal respiration, 1.12 ± 0.45 pmoles/min/10E4 cells for maximal respiration, and 2.86 ± 0.27 pmoles/min/10E4 cells for ATP production). The MCT low-fed group exhibited OCR (6.07 ± 1.72 pmoles/min/10E4 cells for basal respiration, 4.5 ± 1.2 pmoles/min/10E4 cells for maximal respiration, and 5.37 ± 0.93 pmoles/min/10E4 cells for ATP production) that was intermediate between MCT high and AIN-76A control groups (Figure 2B).

Following M1 polarization of M0 BMDM by LPS stimulation, metabolic phenotype was partially affected by MCT intervention compared to the normal diet-fed group (Figure 2C). In detail, OCR in MCT high (9.35 ± 1.57 pmoles/min/10E4 cells for basal respiration, 5.84 ± 0.41 pmoles/10E4 for maximal respiration, and 7.38 ± 0.89 pmoles/min/10E4 cells for ATP production) and MCT low (6.13 ± 1.83 pmoles/min/10E4 cells for basal respiration, 4.08 ± 1.06 pmoles/min/10E4 cells for maximal respiration, and 4.78 ± 1.06 pmoles/min/10E4 cells for ATP production) groups did not significantly differ from the AIN-76A control (3.67 ± 0.47 pmoles/min/10E4 cells for basal respiration, 2.01 ± 0.34 pmoles/min/10E4 cells for maximal respiration, and 4.78 ± 1.06 pmoles/min/10E4 cells for ATP production). However, following dissected quantification of OCR by appropriate drug treatments, maximal respiration of LPS-stimulated (M1) BMDMs in the MCT high-fed group was significantly elevated compared to the normal diet group. Even though the difference in OCR was not statistically significant for basal respiration and ATP production, similar trends to those for maximal respiration were observed (Figure 2D).

Alternatively, M0 BMDMs were polarized to the M2 phenotype by the addition of IL-4 in culture medium. OCRs for MCT oil-fed groups were significantly elevated compared to normal diet-fed controls (Figure 2E). In detail, the MCT high-fed group exhibited significant increments of basal respiration, maximal respiration, and ATP production in M2 BMDMs (15.17 ± 4.03, 11.13 ± 3.62, and 11.97 ± 2.89 pmoles/min/10E4 cells, respectively) compared to the AIN-76A fed control group (3.33 ± 0.54, 0.94 ± 0.25, and 3.47 ± 0.67 pmoles/min/10E4 cells, respectively). Similar to OCR quantification in M0, OCR in the MCT low-fed group (6.6 ± 1.85 pmoles/min/10E4 cells for basal respiration, 4.25 ± 1.54 pmoles/min/10E4 cells for maximal respiration, and 5.67 ± 1.17 pmoles/min/10E4 cells for ATP production) was intermediate between those of MCT and normal diet-fed controls (Figure 2F).

### 3.2. MCT Oil Downregulates Activation Marker Expression on Macrophage Surfaces

**Figure 3:**
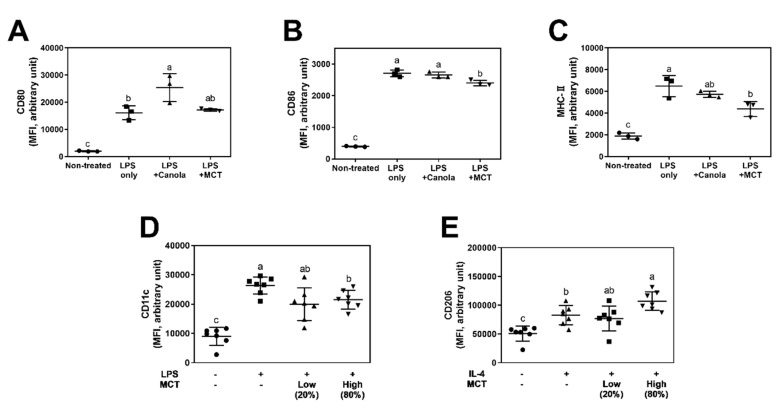
MCT induces macrophages to have anti-inflammatory phenotypes by modulating the expression of surface markers. CD80 (**A**), CD86 (**B**), and MHC-II (**C**) expressions of RAW 264.7 cells were evaluated following oil treatment and/or LPS stimulations (*n* = 3). CD11c (**D**) was evaluated for the M1 marker and CD206 (**E**) for the M2 marker in BMDMs from mice fed with custom diets following LPS and IL-4 stimulation, respectively (*n* = 7). Cells were stained with antibodies specific to surface molecules and analyzed by a flow cytometer. Values are indicated as mean ± SEM, and statistical significance is indicated with different letters (*p* < 0.05).

Given that the mitochondrial respiration of macrophages was modulated by MCT oil through up-regulation of oxygen consumption in vitro (RAW 264.7 cells) and ex vivo (murine BMDMs), we examined whether the regulation of mitochondrial respiration by MCT oil affects the expressions of activation markers on macrophage surfaces. In a cellular model using RAW 264.7 cells, costimulatory molecules, i.e., expression of CD80 (B7-1), CD86 (B7-2), and MHC-II, were determined by specific staining with fluorescence-conjugated antibodies followed by flow cytometry. Canola oil treatment significantly upregulated (*p* < 0.05) CD80 expression in RAW 264.7 cells (25,455 ± 2963 MFI) compared to non-oil-treated controls (16,115 ± 1485 MFI). However, MCT oil-treated cells exhibited comparable expression of CD80 (17,205 ± 253.7 MFI) to non-oil-treated controls (Figure 3A). In addition, MCT oil significantly suppressed the expressions of CD86 (2399 ± 51.46 MFI) and MHC-II molecules (4376 ± 392.5 MFI) compared to non-oil-treated controls (2705 ± 52.93 MFI for CD86 expression and 6490 ± 567.5 MFI for MHC-II expression), while canola oil did not affect the expressions of either CD86 (2652 ± 52.93 MFI) or MHC-II (5726 ± 167.1 MFI) (Figure 3B,C). These results indicate that MCT oil has a potent anti-inflammatory effect on LPS-stimulated M1-like condition in macrophages.

Alternatively, BMDMs isolated from normal diet-fed mice were induced to transform to either M1 or M2 phenotypes by the addition of LPS or IL-4 in culture medium, respectively. Following differentiation, the expressions of CD11c (M1 marker) and CD206 (M2 marker) were assessed by specific antibody staining and flow cytometry. Following LPS stimulation, cells exhibited significantly elevated expressions of CD11c molecules (LPS 26,335 ± 1109 vs. non-LPS 8995 ± 1153 MFI). Cells from MCT high-fed mice in which 80% of total dietary lipid (corn oil) was substituted for MCT tended to suppress the expression of CD11c (21,508 ± 1201 MFI) but the difference was not significant. Of particular interest, cells from MCT low-fed mice, in which MCT replaced 20% of total dietary lipid (corn oil), exhibited significantly down-regulated expression of CD11c (19,981 ± 2132 MFI) compared to AIN-76A normal diet-fed controls (26,335 ± 1109 MFI) (Figure 3D). In IL-4-treated conditions inducing M2 polarization, increase of CD206 expression by IL-4 stimulation in the AIN-76-A diet fed group (76,703 ± 8136 MFI) was significantly augmented by dietary interventions with high levels of MCT (MCT High, 107,127 ± 6089 MFI). Moderate doses of MCT in the MCT low diet group resulted in expression of CD206 (82,656 ± 6323 MFI) that was intermediate between those of AIN-76A and MCT high diet-fed mice, but the difference was not significant (Figure 3E).

Taken together, these results indicate that MCT oil suppresses inflammatory surface markers while elevating anti-inflammatory activation markers of murine macrophages both in vitro and ex vivo.

### 3.3. MCT Oil Induces an Anti-Inflammatory M2-Like Phenotype in Macrophages

**Figure 4:**
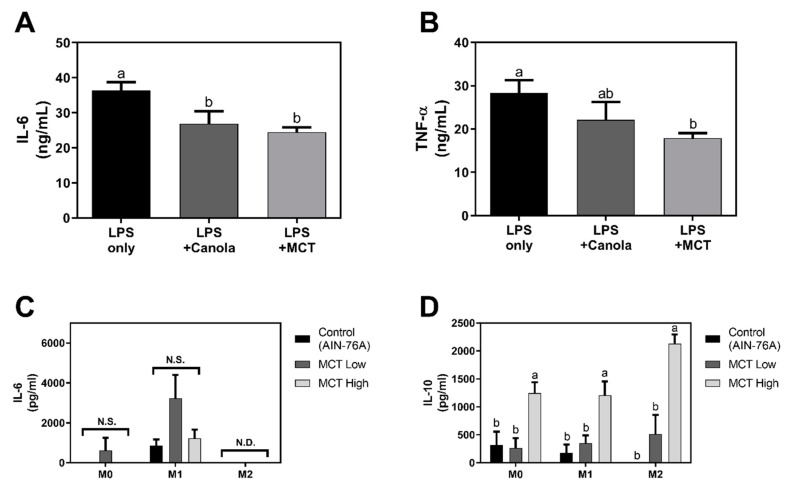
MCT inhibits the production of pro-inflammatory cytokines while enhancing anti-inflammatory cytokine secretion. IL-6 (**A**) and TNF-α (**B**) production of RAW 264.7 macrophages treated with conventional oil samples under LPS-stimulated conditions (*n* = 3). Pro-inflammatory cytokine, IL-6 (**C**), and anti-inflammatory cytokine, IL-10 (**D**) secretion by mice BMDMs under non-polarized (M0), M1, and M2 polarized conditions (*n* = 7). The quantification of cytokines in culture supernatants was determined by ELISA. Data are indicated as mean ± SEM, and statistical significance is presented with different letters (*p* < 0.05, N.S. means no significant difference, N.D. means not detected) within each M0, M1, and M2 column (C,D).

Having found that MCT oil significantly increases M2 surface markers while suppressing M1 surface markers, it was further examined whether the secretion of inflammatory and anti-inflammatory cytokines in macrophages was affected by MCT oil. RAW 264.7 macrophages incubated with MCT oil showed significantly suppressed inflammatory cytokine secretion in LPS-induced conditions. MCT oil significantly down-regulated the production of IL-6 (24.48 ± 1.38 ng/mL) compared to non-oil-treated controls (36.45 ± 2.26 ng/mL). Canola oil, known for its anti-inflammatory and anti-oxidant effects [14], also suppressed the secretion of IL-6 (26.92 ± 2.03 ng/mL) (Figure 4A). MCT oil resulted in similar reductions in TNF-α secretion (17.89 ± 0.67 ng/mL) compared to non-oil-treated controls (28.35 ± 1.69 ng/mL), while canola oil treatment resulted in no significant differences from the control (22.11 ± 2.38 ng/mL) (Figure 4B).

To further assess the dietary effects of MCT on cytokine production, BMDMs (M0) isolated from either AIN-76A control, MCT low, or MCT high diet fed mice were polarized to M1 (LPS) or M2 (IL-4) phenotypes. There was no significant difference in inflammatory cytokine IL-6 production between different diet-fed groups under non-polarized (M0) and LPS-treated (M1) conditions. Following M2 differentiation by IL-4, the secretions of IL-6 was not detected in any diet-fed BMDMs (Figure 4C). In contrast, MCT significantly increased the anti-inflammatory cytokine, IL-10, in all subsets of murine BMDMs ex vivo. The MCT high-fed group exhibited significantly up-regulated IL-10 secretion (1246 ± 194.6 pg/mL) of BMDMs compared to normal diet-fed (318.1 ± 238.3 pg/mL) and MCT low-fed (266 ± 173.9 pg/mL) groups in non-polarized (M0) status. In inflammatory LPS-treated (M1) conditions, IL-10 was also significantly elevated in MCT high-fed mice (1209 ± 245.7 pg/mL) compared to MCT low-fed (351.3 ± 139.6 pg/mL) and normal diet-fed (176.5 ± 148.6 pg/mL) controls. Consistent with previous results, MCT-high fed BMDMs secreted significantly more IL-10 (2135 ± 159.7 pg/mL) compared to other diet-fed groups (Figure 4D). These data suggest that MCT induces M0 and M1 macrophages to transform into M2-like macrophages and enhances anti-inflammatory properties of all subsets of macrophages.

## 4. Discussion

Differentiated macrophages are mainly classified as classically (M1) and alternatively (M2) activated macrophages, which are reported to possess inflammatory and anti-inflammatory properties, respectively [15]. M1 macrophages have inherent microbicidal activity and release pro-inflammatory factors, such as nitric oxides (NO), IL-6, IL-1β, and TNF-α [16]. In contrast, M2 macrophages produce anti-inflammatory mediators such as IL-10 and transforming growth factor β (TGF-β) with anti-parasitic and tissue repair functions [17]. In various experimental approaches, M1 macrophages are activated by LPS or interferon (IFN)-γ, and M2 macrophages are generated in response to stimulation with IL-4 or IL-13 [10,18]. In addition to differences in physiological functions of polarized subsets of macrophages, M1 and M2 macrophages differ in cellular energy producing pathways. In detail, energy metabolism of M1 macrophages is mainly dependent on glycolytic pathway releasing lactate, whereas M2 macrophages rely on fatty acid oxidation (FAO) which provides acetyl-CoA, a substrate for oxidative phosphorylation [19,20]. Accordingly, M1 macrophages were shown to demand lower cellular oxygen than M0 macrophages, whereas M2 macrophages consume oxygen in an increased rate [21]. With this dichotomy in cellular energy metabolism recently found, dietary approaches to modulate energy metabolism and further macrophage function remain poorly investigated.

In this study, we present the novel finding that MCT oil drives macrophages to exhibit anti-inflammatory functions by enhancing mitochondrial respiration. First, well-defined murine macrophage RAW 264.7 cell lines were utilized, even though their polarization to M2 status was not documented to date. MCT oil exhibited no effect on oxygen consumption in a non-stimulated M0-like status (Figure 1A). Interestingly, however, under M1-like inflammatory conditions following LPS stimulation, MCT oil significantly increased OCR for basal respiration, maximal respiration, and ATP production (Figure 1B,C). In this regard, control canola oil had no effect on oxygen demand during inflammatory responses in RAW 264.7 macrophages. The regulation of macrophage OCR by MCT oil was further studied in a dietary model of C57BL/6 mice. Following dietary intervention at low or high doses in which 20% or 80% of corn oil in defined AIN-76A diet was replaced with MCT, respectively, BMDMs were incubated following an established method. BMDMs were further stimulated with either medium (M0), LPS + IFN-γ (M1), or IL-4 + IL-13 (M2), and cellular oxygen demands were assessed to define energy metabolism. M1 polarized BMDMs partially recapitulated the in vitro results by significantly increasing OCR by MCT oil intervention in maximal respiration (Figure 2C,D), while the consistent trends were observed in basal respiration and ATP production. Interestingly, however, M0 (Figure 2A,B) and M2 (Figure 2E,F) BMDMs from MCT oil-fed mice exhibited significantly elevated OCR for basal respiration, maximal respiration, and ATP production. These results suggest that MCT oil may affect the intracellular physiology, including energy metabolism, of non-polarized (M0) macrophages and ease their conversion towards M2-like macrophages in an appropriate cue.

Following investigation on energy metabolism in macrophages, the effect of MCT oils on cellular functions were studied. RAW 264.7 cells are well defined for their activation markers, CD80, CD86, and MHC II following LPS-induced inflammatory signaling. Culture of cells in the presence of MCT suppressed their expression on the cell surfaces as determined by immunostaining of fluorescent specific antibodies and flowcytometric analysis (Figure 3A–C). Similarly, dietary intervention of MCT oil also suppressed the expression of CD11c (Figure 3D), a typical surface marker for M1 macrophages [22], in LPS+IFN-γ induced M1 BMDMs. MCT oil further suppressed the production of inflammatory cytokine IL-6 and TNF-α in LPS activated RAW 264.7 cells (Figure 4A,B), but LPS + IFN-γ polarized M1 BMDMs exhibited no difference in IL-6 secretion ex vivo (Figure 4C). In this regard, medium-chain fatty acids were previously demonstrated to suppress NF-κB and ERK signaling pathways in mature osteoclasts [23]. It is noteworthy that IL-6 and TNF-α are the downstream products of that signaling. Similarly, inhibitory trends leading to down-regulation of the transcriptions of iNOS and COX-2 mRNA, both pro-inflammatory downstream genes of NF-κB and ERK signaling, were exhibited by MCT oil (Supplementary Figure S2). In M2 cells it was reported that suppressed iNOS transcription leads to elevated arginine, which is further metabolized to proline required for tissue remodeling and repair [24].

In addition to the suppression of inflammatory responses of macrophages by MCT both in cellular and animal models, its effect on M2 polarized cells were investigated in vivo. In IL-4 + IL-13 polarized M2 BMDMs, MCT oil enhanced the expression of CD206, a delineative activation marker of M2 macrophages [22,25] (Figure 3E). Surprisingly, MCT oil further increased the secretion of IL-10, an anti-inflammatory cytokine, in M0, M1, and M2 polarized BMDMs (Figure 4D). The discrepancy between the cellular vs. animal model may come from the physiological differences of macrophages. As mentioned above, immortal RAW 264.7 cells are derived from mouse peritoneal macrophages [26] and inclined to M1 responses within 24 h stimulation of LPS. In contrast, LPS + IFN-γ polarized M1 BMDMs are developed from bone-marrow progenitor cells through naïve M0 induction in a culture plate for 7 days. In addition, the difference in MCT treatment should be noted. RAW 264.7 cells are treated with MCT in the presence of LPS in culture media, where mice are consuming MCT as a dietary lipids and no oil is supplied during BMDM polarization. Nevertheless, the results are complementary to each other indicating that MCT not only suppresses inflammatory responses in M1 status, but also actively upregulates the switch of macrophage function towards M2-like repolarization. Our findings that MCT enhanced anti-inflammatory responses of macrophages via up-regulating mitochondrial respirations are in accordance with previous reports, in which increased energy expenditure by medium-chain fatty acids (MCFAs) drives macrophages to exhibit anti-obesity and anti-inflammatory phenotypes [9,27].

Such reprogramming of macrophage energy metabolism and consequent anti-inflammatory effects of MCT are postulated to originate from molecular structures of the medium-chain fatty acids, which contain 6–12 carbons. Indeed, MCT oil used in the current study mainly contains caprylic (C8:0) and capric (C10:0) acids, which were previously reported to inhibit pro-inflammatory cytokines in rats and mice [28,29]. In the digestive tract, the medium-chain fatty acids of MCT are degraded into free fatty acids by lipase [30]. Liberated MCFAs are effectively transported to liver via the hepatic portal vein directly and penetrate through mitochondrial inner-membranes, resulting in rapid oxidization to acetyl-CoA through β-oxidation [31,32]. In contrast, long-chain fatty acids (LCFAs) such as stearic (16:0) or palmitic (18:0) acid require carnitine-dependent transport due to their lower hydrophilicity [33,34]. Therefore, elevated acetyl-CoA by oxidation of MCFAs facilitates oxidative phosphorylation resulting in increments of OCR in macrophages.

It should be noted, however, that no effect of FAO on macrophage polarization was also reported [35], in which the contribution of FAO was assessed by either inhibition of carnitine-palmitoyl transferases or knock-out of fatty acid transport proteins. Alternative mechanisms by which fatty acids may affect macrophage phenotypes are also suggested [36,37], and the actual molecular mechanisms remain unproven. Those studies are focused on the role of LCFAs and their oxidation, but the current study is novel in the use of MCFAs for macrophage polarization.

As for the dietary regulation of MCT on immune function, it was recently found that MCT activate human neutrophils, exemplified by effects on respiratory burst [38], expression of adhesion and degranulation markers [39], and functional outcomes such as migration [40] and phagocytosis [41]. Taken together, our results demonstrate that MCT, which are enriched in coconut and palm oils, not only enhance the anti-inflammatory properties of non-polarized or M2 macrophages, but also exhibits the ability to shunt M1 macrophages to M2-like status by reprogramming mitochondrial respiration. An apparent limit of the current study is that antioxidant supplementation in the oils was not counted for the experimental readouts. To validate our findings, further studies are required to elucidate the molecular mechanisms of medium-chain fatty acids for phenotypic switching of M1 to M2 macrophages.

## Figures and Tables

**Table 1:** Custom diet composition.

Nutrient	Normal (AIN-76A) grams/kg	High-MCT grams/kg	Low-MCT grams/kg
Casein	200	200	200
dl-methionine	3	3	3
Corn Starch	150	150	150
Sucrose	500	500	500
Cellulose	50	50	50
Corn oil	50	10	40
Medium-chain fatty acid oil	0	40	10
Mineral mix	35	35	35
Vitamin mix	10	10	10
Choline bitartrate	2	2	2
Total	1000	1000	1000

## Supplementary Materials

The following are available online at https://www.mdpi.com/2304-8158/8/11/553/s1, Figure S1: Mouse body weight, Figure S2: Effects of MCT on transcription of pro-inflammatory enzymes in RAW 264.7 macrophages.
