# Supplementary material for: Medium Chain Triglyceride (MCT) Oil Affects the Immunophenotype via Reprogramming of Mitochondrial Respiration in Murine Macrophages
Source: Foods. 2019 Nov 5;8(11):553. doi: 10.3390/foods8110553 (PMC6915711; doi:10.3390/foods8110553)
Supplement: Supplementary file 1 [file foods-08-00553-s001.pdf]

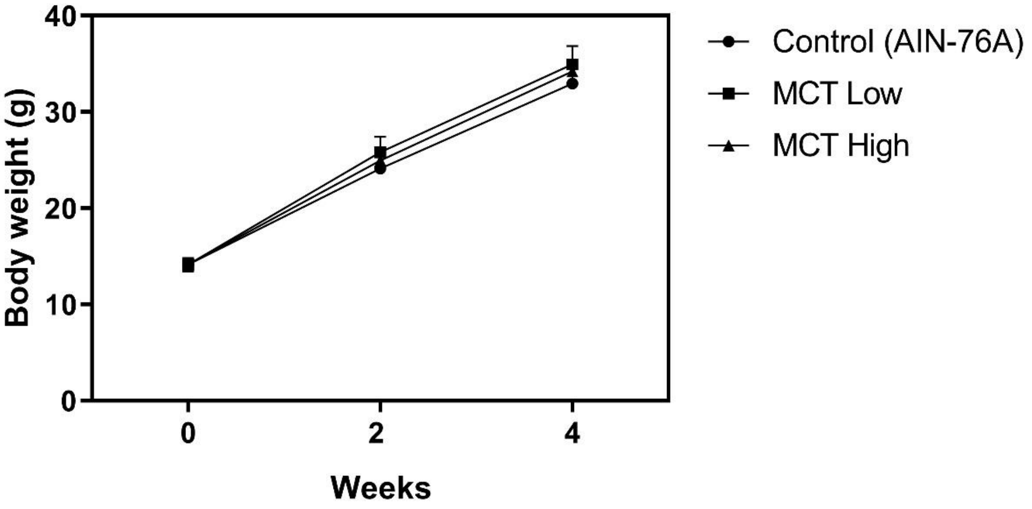

Figure S1. Mouse body weight. Each mouse was fed a custom diets and body weight was calculated every 2 weeks. Data are presented as mean±SD (n = 7).

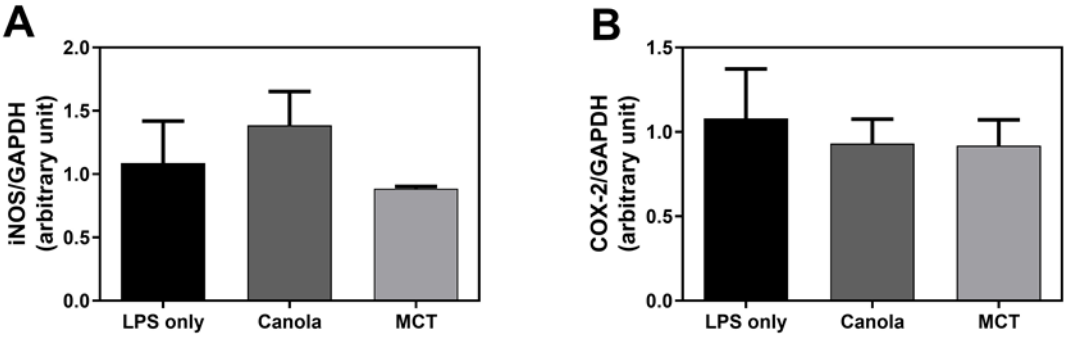

Figure S2. Effects of MCT on transcription of pro-inflammatory enzymes in RAW 264.7 macrophages. iNOS (**A**) and COX-2 (**B**) mRNA transcription following oil treatment and LPS stimulation. The relative levels of mRNA were determined by qRT-PCR and calculated relative to GAPDH by the Δ ΔCt method. Data are presented as mean±SEM (n = 3).
